# Supplementary material for: OpenMM 7: Rapid development of high performance algorithms for molecular dynamics
Source: PLoS Comput Biol. 2017 Jul 26;13(7):e1005659. doi: 10.1371/journal.pcbi.1005659 (PMC5549999; doi:10.1371/journal.pcbi.1005659)
Supplement: S1 Source Code — (ZIP) [file pcbi.1005659.s002.zip › OpenMM-7.0.1-Source/docs/api-c++/generated/OpenMM.Platform.html]

Platform — OpenMM @OPENMM\_MAJOR\_VERSION@.@OPENMM\_MINOR\_VERSION@ documentation


# Platform¶

*class* `OpenMM::``Platform`¶
:   A `Platform` defines an implementation of all the kernels needed to perform some calculation. More precisely, a `Platform` object acts as a registry for a set of KernelFactory objects which together implement the kernels. The `Platform` class, in turn, provides a static registry of all available `Platform` objects.

    To get a `Platform` object, call

    ```
    Platform& platform = Platform::findPlatform(kernelNames);
    ```

    passing in the names of all kernels that will be required for the calculation you plan to perform. It will return the fastest available `Platform` which provides implementations of all the specified kernels. You can then call `createKernel()` to construct particular kernels as needed.

    Methods

    |  |  |
    | --- | --- |
    | `~Platform` |  |
    | `getName` | Get the name of this platform. |
    | `getSpeed` | Get an estimate of how fast this `Platform` class is. |
    | `supportsDoublePrecision` | Get whether this `Platform` supports double precision arithmetic. |
    | `getPropertyNames` | Get the names of all Platform-specific properties this `Platform` supports. |
    | `getPropertyValue` | Get the value of a Platform-specific property for a `Context`. |
    | `setPropertyValue` | Set the value of a Platform-specific property for a `Context`. |
    | `getPropertyDefaultValue` | Get the default value of a Platform-specific property. |
    | `setPropertyDefaultValue` | Set the default value of a Platform-specific property. |
    | `contextCreated` | This is called whenever a new `Context` is created. |
    | `contextDestroyed` | This is called whenever a `Context` is deleted. |
    | `registerKernelFactory` | Register a KernelFactory which should be used to create Kernels with a particular name. |
    | `supportsKernels` | Determine whether this Platforms provides implementations of a set of kernels. |
    | `createKernel` | Create a Kernel object. |

    `~Platform`()¶

    *const* std::string &`getName`() *const* = 0¶
    :   Get the name of this platform. This should be a unique identifier which can be used to recognized it.

    double `getSpeed`() *const* = 0¶
    :   Get an estimate of how fast this `Platform` class is. This need not be precise. It only is expected to return an order or magnitude estimate of the relative performance of different `Platform` classes. An unoptimized reference implementation should return 1.0, and all other Platforms should return a larger value that is an estimate of how many times faster they are than the reference implementation.

    bool `supportsDoublePrecision`() *const* = 0¶
    :   Get whether this `Platform` supports double precision arithmetic. If this returns false, the platform is permitted to represent double precision values internally as single precision.

        Deprecated

        This method is not well defined, and is too simplistic to describe the actual behavior of some Platforms, such as ones that offer multiple precision modes. It will be removed in a future release.

    *const* std::vector<std::string> &`getPropertyNames`() *const*¶
    :   Get the names of all Platform-specific properties this `Platform` supports.

    *const* std::string &`getPropertyValue`(*const* Context &*context*, *const* std::string &*property*) *const*¶
    :   Get the value of a Platform-specific property for a `Context`.

        |  |  |
        | --- | --- |
        | Parameters: |  |

        - **context** – the `Context` for which to get the property
        - **property** – the name of the property to get

        |  |  |
        | --- | --- |
        | Returns: | the value of the property |

    void `setPropertyValue`(Context &*context*, *const* std::string &*property*, *const* std::string &*value*) *const*¶
    :   Set the value of a Platform-specific property for a `Context`.

        |  |  |
        | --- | --- |
        | Parameters: |  |

        - **context** – the `Context` for which to set the property
        - **property** – the name of the property to set
        - **value** – the value to set for the property

    *const* std::string &`getPropertyDefaultValue`(*const* std::string &*property*) *const*¶
    :   Get the default value of a Platform-specific property. This is the value that will be used for newly created Contexts.

        |  |  |
        | --- | --- |
        | Parameters: |  |

        - **property** – the name of the property to get

        |  |  |
        | --- | --- |
        | Returns: | the default value of the property |

    void `setPropertyDefaultValue`(*const* std::string &*property*, *const* std::string &*value*)¶
    :   Set the default value of a Platform-specific property. This is the value that will be used for newly created Contexts.

        |  |  |
        | --- | --- |
        | Parameters: |  |

        - **property** – the name of the property to set
        - **value** – the value to set for the property

    void `contextCreated`(ContextImpl &*context*, *const* std::map<std::string, std::string> &*properties*) *const*¶
    :   This is called whenever a new `Context` is created. It gives the `Platform` a chance to initialize the context and store platform-specific data in it.

        |  |  |
        | --- | --- |
        | Parameters: |  |

        - **context** – the newly created context
        - **properties** – a set of values for platform-specific properties. Keys are the property names.

    void `contextDestroyed`(ContextImpl &*context*) *const*¶
    :   This is called whenever a `Context` is deleted. It gives the `Platform` a chance to clean up any platform-specific data that was stored in it.

    void `registerKernelFactory`(*const* std::string &*name*, KernelFactory \**factory*)¶
    :   Register a KernelFactory which should be used to create Kernels with a particular name. The `Platform` takes over ownership of the factory, and will delete it when the `Platform` itself is deleted.

        |  |  |
        | --- | --- |
        | Parameters: |  |

        - **name** – the kernel name for which the factory should be used
        - **factory** – the factory to use for creating Kernels with the specified name

    bool `supportsKernels`(*const* std::vector<std::string> &*kernelNames*) *const*¶
    :   Determine whether this Platforms provides implementations of a set of kernels.

        |  |  |
        | --- | --- |
        | Parameters: |  |

        - **kernelNames** – the names of the kernels of interests

        |  |  |
        | --- | --- |
        | Returns: | true if this `Platform` provides implementations of all the kernels in the list, false if there are any which it does not support |

    Kernel `createKernel`(*const* std::string &*name*, ContextImpl &*context*) *const*¶
    :   Create a Kernel object. If you call this method multiple times for different contexts with the same name, the returned Kernels are independent and do not interact with each other. This means that it is possible to have multiple simulations in progress at one time without them interfering.

        If no KernelFactory has been registered for the specified name, this will throw an exception.

        |  |  |
        | --- | --- |
        | Parameters: |  |

        - **name** – the name of the Kernel to get
        - **context** – the context for which to create a Kernel

        |  |  |
        | --- | --- |
        | Returns: | a newly created Kernel object |

    void `registerPlatform`(Platform \**platform*)¶
    :   Register a new `Platform`.

    int `getNumPlatforms`()¶
    :   Get the number of Platforms that have been registered.

    Platform &`getPlatform`(int *index*)¶
    :   Get a registered `Platform` by index.

    std::vector<std::string> `getPluginLoadFailures`()¶
    :   Get any failures caused during the last call to loadPluginsFromDirectory

    Platform &`getPlatformByName`(*const* std::string &*name*)¶
    :   Get the registered `Platform` with a particular name. If no `Platform` with that name has been registered, this throws an exception.

    Platform &`findPlatform`(*const* std::vector<std::string> &*kernelNames*)¶
    :   Find a `Platform` which can be used to perform a calculation.

        |  |  |
        | --- | --- |
        | Parameters: |  |

        - **kernelNames** – the names of all kernels which will be needed for the calculation

        |  |  |
        | --- | --- |
        | Returns: | the fastest registered `Platform` which supports all of the requested kernels. If no `Platform` exists which supports all of them, this will throw an exception. |

    void `loadPluginLibrary`(*const* std::string &*file*)¶
    :   Load a dynamic library (DLL) which contains an OpenMM plugin. Typically, each `Platform` is distributed as a separate dynamic library. This method can then be called at runtime to load each available library. Each library should contain an initializer function to register any Platforms and KernelFactories that it contains.

        If the file does not exist or cannot be loaded, an exception is thrown.

        |  |  |
        | --- | --- |
        | Parameters: |  |

        - **file** – the path to the dynamic library file. This is interpreted using the operating system’s rules for loading libraries. Typically it may be either an absolute path or relative to a set of standard locations.

    std::vector<std::string> `loadPluginsFromDirectory`(*const* std::string &*directory*)¶
    :   Load multiple dynamic libraries (DLLs) which contain OpenMM plugins from a single directory. This method loops over every file contained in the specified directory and calls `loadPluginLibrary()` for each one. If an error occurs while trying to load a particular file, that file is simply ignored. You can retrieve a list of all such errors by calling `getPluginLoadFailures()`.

        |  |  |
        | --- | --- |
        | Parameters: |  |

        - **directory** – the path to the directory containing libraries to load

        |  |  |
        | --- | --- |
        | Returns: | the names of all files which were successfully loaded as libraries |

    *const* std::string &`getDefaultPluginsDirectory`()¶
    :   Get the default directory from which to load plugins. If the environment variable OPENMM\_PLUGIN\_DIR is set, this returns its value. Otherwise, it returns a platform specific default location.

        |  |  |
        | --- | --- |
        | Returns: | the path to the default plugin directory |

    *const* std::string &`getOpenMMVersion`()¶
    :   Get a string containing the version number of the OpenMM library.

High performance molecular simulation on GPUs


### Navigation

- Core Objects
- Forces
- Integrators
- Extras

©2015, Stanford University and the Authors.
|
Powered by Sphinx 1.3.1
& Alabaster 0.7.7
|
Page source
